# A time-delayed model for the spread of COVID-19 with vaccination

**DOI:** 10.1038/s41598-022-23822-5

**Published:** 2022-11-13

**Authors:** Salma M. Al-Tuwairqi, Sara K. Al-Harbi

**Affiliations:** Mathematics Department, King Abdulaziz University, Jeddah, Saudi Arabia

**Keywords:** Mathematics and computing, Applied mathematics

## Abstract

A mathematical model is presented in this paper to investigate the effects of time delay in vaccine production on COVID-19 spread. The model is analyzed qualitatively and numerically. The qualitative analysis indicates that the system variables are non-negative, bounded, and biologically meaningful. Moreover, the model has produced two equilibrium points: the free equilibrium point, which can exist without conditions, and the endemic equilibrium point, which can exist if the control reproduction number, ${\mathcal {R}}_c$, is not less than one. In addition, the local stability of the equilibrium points is investigated and agrees with the numerical analysis results. Finally, a sensitivity analysis is conducted for ${\mathcal {R}}_c$. In particular, we examine the effect of the vaccine’s time delay, vaccine rate, and vaccine efficiency on the model dynamics.

## Introduction

Vaccination is a control measure to combat infectious diseases and reach herd immunity. It is through vaccination that smallpox disease was eliminated from the world^1^. Since the COVID-19 pandemic began, significant efforts have been made to develop a vaccine against the SARS-CoV-2 virus worldwide. Developing a vaccine for a new disease takes time. First, it is necessary to identify the nature of the virus and how it works. Second, after the drug is chemically produced, it goes through the stage of laboratory tests and animal experiments. If the trials give positive results, the vaccine enters the phase of clinical trials, which include phases to ensure the safety and effectiveness of the vaccine and to know the appropriate dose and side effects. After the regulatory agencies approve the vaccine, it moves to the manufacturing and distribution phase^2,3^.

Many studies have mathematically examined the impact of the COVID-19 vaccine in controlling the spread of the disease. For example, Annas et al.^4^ constructed an SEIR model with the vaccine as a parameter in the model. They investigated the model analytically and numerically to predict the number of COVID-19 cases in Indonesia if vaccination is implemented. Alshammari and Akyildiz^5^ modeled the epidemic of COVID-19 dynamics using the SIR model with nonstandard nonlinear incidence and recovery rates. They presented two models with and without the vaccine, where they also considered the vaccine as a model parameter. The study showed that the vaccination term model gives a better fit to the real data of Saudi Arabia. On the other hand, Ghostine et al.^6^ presented the COVID-19 model with a bilinear incidence rate, exhibiting the vaccinated population as a separate compartment. They applied the model to data from Saudi Arabia and investigated the effect of vaccination on controlling the disease. Moreover, Rana and Sharma^7^ created a seven compartments model by adding the vaccinated and hospitalized populations to the traditional susceptible, exposed, symptomatic, asymptomatic, and recovered populations. The model was fitted to data from four regions of India and Russia. They explored the effect of lockdown, vaccination, and drug treatment as control measures. Another model incorporating the compartment of the vaccinated population is given in^8^, where they discussed the effect of optimal vaccination and social distancing on COVID-19 in India. The previous studies agreed with the clinical study in^9^, where they confirmed that the COVID-19 vaccine reduced the mortality and the need for intensive care units for vaccinated individuals in Dammam, Saudi Arabia.

Time-delayed differential equations have been utilized in modeling the spread of COVID-19. It was used to describe the characteristics of COVID-19, such as incubation and latent period, recovery time, diagnosis time, and immune response. Cakan, in^10^, proposed an SEIR model representing the latent period of COVID-19 as a time delay parameter. The model investigates the capacity of health care by assuming the variability of recovery and death rates due to COVID-19. Barman and Mishra^11^ also modeled the incubation period in COVID-19 disease as a time delay parameter; however, they included the asymptomatic population in the dynamics of the model. Yang and Zhang^12^ described the propagation dynamics of COVID-19 using the SEIQR model with two-time delays. They considered the delay in time for an exposed individual to convert to an infected individual. Also, they incorporated in the model recovery time delay for exposed, infected, and quarantined individuals. The model produced a unique endemic equilibrium point where its local stability depends on the time delays. On the other hand, Lu et al.^13^ modeled the spread of COVID-19, excluding the exposed population, using the SIQR model but regarding the recovery time delay for infected individuals. Two equilibrium points were accomplished, the virus free and the endemic equilibrium, where again, stability is determined according to the time delay values. Radha and Balamuralitharan^14^ considered the time delay for the immune system to respond to the transmission dynamics of COVID-19. They used an SEIR model and attained three equilibrium points that are stable under specific criteria. Finally, Yang^15^ presented a COVID-19 model considering the delayed time for an infected individual to be diagnosed and quarantined in hospitals. If the time delay is long, COVID-19 is not controllable. They concluded that home isolation and social distancing aid in containing the disease.

As for models representing time-delayed dynamics of COVID-19 with vaccination measures, Zhai et al.^16^ proposed an SEIR model with a time delay for infected individuals to be infectious. They incorporated a vaccination strategy that switches when the basic reproduction number exceeds one. They tested the model during the second outbreak of COVId-19 in Italy. Moreover, Amaku et al.^17^ examined the effect of the delay in COVID-19 vaccination on the number of infected cases in Brazil. Their model included a compartment of vaccinated individuals and a compartment of vaccinated individuals who failed to be immunized. They concluded that severe consequences would occur due to the vaccination delay.

To our knowledge, no mathematical model for COVID-19 dynamics examines the delay time in vaccine production. This work aims to build a mathematical model using time-delayed differential equations to discuss the impact of the delay in producing the COVID-19 vaccine and the effect of vaccination rate and its efficiency on COVID-19. The paper is outlined as follows. In section “Mathematical model”, we formulate the model and prove it is epidemiologically appropriate. In section “Qualitative analysis”, we show the model’s qualitative analysis, the equilibrium points’ existence, and their local stability. Moreover, we compute the formula of the control reproduction number, ${\mathcal {R}}_c$. Then, we demonstrate in section “Numerical analysis” the numerical analysis that affirms its agreement with the qualitative analysis and analyzes the effect of the time delay, vaccination rate, and vaccine efficacy. Furthermore, we present the sensitivity analysis for the threshold quantity ${\mathcal {R}}_c$.

## Mathematical model

**Figure 1:**
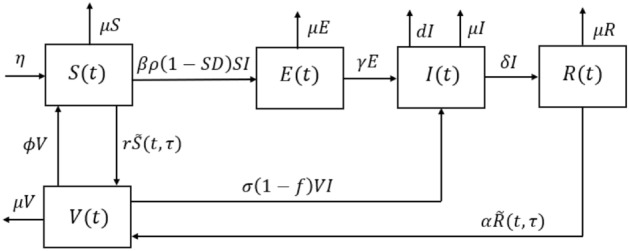
Flowchart of the model.

We divide the total population size, *N*, into five classes: susceptible, exposed, infected, recovered, and vaccinated, which is denoted by $S(t),\ E(t),\ I(t),\ R(t)$, and *V*(*t*), respectively. Each class describes the number of individuals at a time *t*. Therefore, all variables are non-negative. Individuals in the susceptible class move to the vaccinated class at a vaccination rate of *r*. Also, they transfer to the exposed class after connection with infected individuals with a transmission rate of $\beta$. The transmission of COVID-19 is affected by the preventive measures, the lockdown ($\rho \in (0,1]$) and the social distancing ($SD\in [0,1)$). At the end of the incubation period, the exposed individuals transfer to the infected class at a rate of $\gamma$. Then, individuals in the infected class recover at the rate of $\delta$ or die due to COVID-19 at the rate of *d*. A percentage $\alpha$ of individuals in the recovered class receive the vaccine and move to the vaccinated class. After receiving the vaccine, individuals may lose their immunity from COVID-19 and move to the susceptible class at a rate of $\phi$. Infection after a vaccine is affected by the efficiency ratio of vaccine *f*, which differs from one vaccine to another. Depending on the vaccine’s efficiency, vaccinated individuals move to the infected class at a rate of $\sigma$ due to contact with infected individuals. Moreover, we assume that all newborns are included in the susceptible class at a birth rate $\eta$, and the natural death rate from all classes is $\mu$. The dynamics of the model are illustrated in Fig. 1.

The following nonlinear system of delayed differential equations governs the dynamics of the model:

1 $$\begin{aligned} \begin{aligned} \frac{dS}{dt}&=\eta -\beta \rho (1-SD)SI-r{\tilde{S}}(t,\tau )+\phi V-\mu S, \\ \frac{dE}{dt}&=\beta \rho (1-SD)SI-(\gamma +\mu )E,\\ \frac{dI}{dt}&=\gamma E+\sigma (1-f)VI-(\delta +d+\mu )I,\\ \frac{dR}{dt}&=\delta I-\alpha {\tilde{R}}(t,\tau )-\mu R,\\ \frac{dV}{dt}&=r{\tilde{S}}(t,\tau )+\alpha {\tilde{R}}(t,\tau )-\sigma (1-f)VI-(\phi +\mu )V. \end{aligned} \end{aligned}$$

All the parameters in system (1) are non-negative. The parameter $\tau$ is the time delay in producing the vaccine for COVID-19. The ${\tilde{S}}(t,\tau )$ and ${\tilde{R}}(t,\tau )$ terms describe the susceptible and recovered individuals who did not receive the vaccine at the time $t-\tau$, respectively. Moreover, we assume that the rate of change of ${\tilde{S}}(t,\tau )$ decreases at a rate of $\mu$ as in^10^, which is given by the following equation

2 $$\begin{aligned} \Big (\frac{\partial }{\partial t}+\frac{\partial }{\partial \tau }\Big ){\tilde{S}}(t,\tau )= -\mu {\tilde{S}}(t,\tau ). \end{aligned}$$

Since the expression ${\tilde{S}}(t,0)$ indicates the number of susceptible individuals at time *t*, which is equal to *S*(*t*), therefore, consider the first order partial differential equation (2) with the boundary condition, ${\tilde{S}}(t,0)=S(t).$

Similarly, we assume that the rate of change of ${\tilde{R}}(t,\tau )$ decreases at a rate of $\mu$ and given by the following equation

3 $$\begin{aligned} \Big (\frac{\partial }{\partial t}+\frac{\partial }{\partial \tau }\Big ) {\tilde{R}}(t,\tau )=-\mu {\tilde{R}}(t,\tau ). \end{aligned}$$

The expression ${\tilde{R}}(t,0)$ indicates the number of recovered individuals at time *t*, which is equal to $\delta I(t)$. Therefore, consider the equation (3) with the boundary condition, ${\tilde{R}}(t,0)=\delta I(t).$

We use the method of characteristics to solve the first-order partial differential equations^18^. From (2), the characteristic equation is

$$\begin{aligned} dt=d\tau =\frac{d{\tilde{S}}(t,\tau )}{-\mu {\tilde{S}}(t,\tau )}. \end{aligned}$$

First, we solve the equation

$$\begin{aligned} -\mu d\tau =\frac{d{\tilde{S}}}{{\tilde{S}}}. \end{aligned}$$

Integrating both sides, we get $-\mu \tau +A_{1}=\ln {{\tilde{S}}(t,\tau )}$, where $A_{1}$ is the constant of integration and $A_{1}=\ln {{\tilde{S}}(t,\tau )}+\mu \tau$. Then, solve the equation

$$\begin{aligned} dt=d\tau . \end{aligned}$$

We get $B_{1}=t-\tau$. Using the formula $A_{1}=F(B_{1})$, we obtain

4 $$\begin{aligned} \ln {{\tilde{S}}(t,\tau )}+\mu \tau =F(t-\tau ). \end{aligned}$$

Substitute $\tau =0$ and use the boundary condition ${\tilde{S}}(t,0)=S(t)$, we get $\ln {S(t)}=F(t)$. Then equation (4) becomes

$$\begin{aligned} \begin{aligned} \ln {{\tilde{S}}(t,\tau )}&=F(t-\tau )-\mu \tau, \\ \ln {{\tilde{S}}(t,\tau )}&=\ln {S(t-\tau )}-\mu \tau. \end{aligned} \end{aligned}$$

Hence, the solution of equation (2) is

5 $$\begin{aligned} {\tilde{S}}(t,\tau )=S(t-\tau ) e^{-\mu \tau }. \end{aligned}$$

Similarly the solution of equation (3) is

6 $$\begin{aligned} {\tilde{R}}(t,\tau )=\delta I(t-\tau ) e^{-\mu \tau }. \end{aligned}$$

Then, model (1) can be rewritten as follows:

7 $$\begin{aligned} \begin{aligned} \frac{dS}{dt}&=\eta -\beta \rho (1-SD)SI-r S(t-\tau )e^{-\mu \tau }+\phi V-\mu S, \\ \frac{dE}{dt}&=\beta \rho (1-SD)SI-(\gamma +\mu )E,\\ \frac{dI}{dt}&=\gamma E+\sigma (1-f)VI-(\delta +d+\mu )I,\\ \frac{dR}{dt}&=\delta I-\alpha \delta I(t-\tau )e^{-\mu \tau }-\mu R,\\ \frac{dV}{dt}&=r S(t-\tau )e^{-\mu \tau }+\alpha \delta I(t-\tau ) e^{-\mu \tau }-\sigma (1-f)VI-(\phi +\mu )V. \end{aligned} \end{aligned}$$

The term $e^{-\mu \tau }$ is the probability that the individual survives during the delay period in the relevant class, susceptible or recovered. The initial functions of system (7) are

8 $$\begin{aligned} \begin{aligned} S(\zeta )&= \varphi _{1}(\zeta ), \quad&E(\zeta )= \varphi _{2}(\zeta ),\\ I(\zeta )&= \varphi _{3}(\zeta ), \quad&R(\zeta )= \varphi _{4}(\zeta ),\\ V(\zeta )&= \varphi _{5}(\zeta ), \quad&\zeta \in [-\tau ,0], \end{aligned} \end{aligned}$$

where, $\varphi _{i}(\zeta )\ge 0\ (i=1,2,\ldots ,5),$ and $(\varphi _{1},\ \varphi _{2},\ldots ,\ \varphi _{5})\in {\mathcal {C}}([-\tau ,0],{\mathbb {R}}_{\ge 0}^{5})$. Where, ${\mathcal {C}}$ is the Banach space of continuous function mapping the interval $[-\tau ,0]$ into ${\mathbb {R}}_{\ge 0}^{5}$. By the theorem of delay differential equation^19^, system (7) has a unique solution which satisfies the initial functions (8).

### Theorem 1

If $(S,E,I,R,V)\in {\mathbb {R}}_{\ge 0}^{5}$ is a solution of system (7) with the initial functions (8), then the set

$$\begin{aligned} \Omega =\bigg \{(S,E,I,R,V)\in {\mathbb {R}}_{\ge 0}^{5}:0\le N\le \frac{\eta }{\mu } \bigg \} \end{aligned}$$

is positively invariant for system (7).

### Proof

Let $(S(0),E(0),I(0),R(0),V(0))\in \Omega$. From the equations of system (7), we have

$$\begin{aligned} \frac{dS}{dt}\Big |_{S=0}&=\eta +\phi V\ge 0, \ \text {for all}\ V\ge 0, \\ \frac{dE}{dt}\Big |_{E=0}&=\beta \rho (1-SD)SI\ge 0, \ \text {for all}\ S,\ I\ge 0,\\ \frac{dI}{dt}\Big |_{I=0}&=\gamma E\ge 0, \ \text {for all}\ E\ge 0,\\ \frac{dR}{dt}\Big |_{R=0}&=\delta I(1-\alpha )\ge 0, \ \text {for all}\ I\ge 0,\ \tau =0,\\ \frac{dR}{dt}\Big |_{R=0}&\ge \delta I(1-\alpha e^{-\mu \tau })\ge 0, \ \text {for all}\ I\ge 0,\ \tau >0,\\ \frac{dV}{dt}\Big |_{V=0}&=(r S(t-\tau )+\alpha \delta I(t-\tau )) e^{-\mu \tau }\ge 0, \ \text {for all}\ S,\ I\ge 0. \end{aligned}$$

Note that $\alpha <1$ and $e^{-\mu \tau }<1$. Thus, for all $t\ge 0,$ all non-negative solutions remain non-negative. By combining all the equations of system (7), we obtain

$$\begin{aligned} \frac{dN}{dt}=\eta -d I-\mu N\le \eta -\mu N. \end{aligned}$$

Then, solving the above inequality by the integrating factor method^20^, we have

$$\begin{aligned} \frac{d}{du}\left[ e^{\mu u}N(u)\right] \le \eta e^{\mu u}. \end{aligned}$$

Integrating both sides over the time interval [0, *t*], we get

$$\begin{aligned} N(t)\le \frac{\eta }{\mu }+\left[ N(0)-\frac{\eta }{\mu } \right] e^{-\mu t}. \end{aligned}$$

Therefore,

$$\begin{aligned} \lim _{t \rightarrow \infty }Sup\left[ N(t)\right] \le \frac{\eta }{\mu }. \end{aligned}$$

Hence, all solutions of system (7) are bounded and non-negative for all $t\ge 0$. Thus, $\Omega$ is positively invariant.

## Qualitative analysis

In this section, we find the two equilibria for system (7) and compute the control reproduction number. Furthermore, we analyze the local stability behavior of the equilibrium points by using the linearization method^21^.

### Equilibrium points and basic reproduction number

Before finding the equilibrium points for system (7), we assume that

$$\begin{aligned} \lim _{t \rightarrow \infty }S(t)&=\lim _{t \rightarrow \infty }S(t-\tau ), \\ \lim _{t \rightarrow \infty }I(t)&=\lim _{t \rightarrow \infty }I(t-\tau ). \end{aligned}$$

Then, we set the rates in (7) to zero and solve the following system

9 $$\begin{aligned} \eta -\beta \rho (1-SD)SI-r Se^{-\mu \tau }+\phi V-\mu S&=0, \nonumber \\ \beta \rho (1-SD)SI-(\gamma +\mu )E&=0, \nonumber \\ \gamma E+\sigma (1-f)VI-(\delta +d+\mu )I&=0, \nonumber \\ \delta I-\alpha \delta I e^{-\mu \tau }-\mu R&=0, \nonumber \\ r S e^{-\mu \tau }+\alpha \delta I e^{-\mu \tau }-\sigma (1-f)VI-(\phi +\mu )V&=0. \end{aligned}$$

System (9) indicates that there are two equilibrium points. The COVID-19 free equilibrium, $P_0$, which is always present and obtained by setting $E=I=0$, that is,

$$\begin{aligned} P_{0}= \left( \frac{\eta (\phi +\mu )}{\mu re^{-\mu \tau }+\mu (\phi +\mu )},0,0,0,\frac{\eta re^{-\mu \tau }}{\mu re^{-\mu \tau }+\mu (\phi +\mu )} \right) . \end{aligned}$$

To find the COVID-19 endemic equilibrium, $P_{1}$, we solve the fourth equation of system (9) for *R*, that is,

$$\begin{aligned} R=\frac{\delta (1-\alpha e^{-\mu \tau })}{\mu }\ I. \end{aligned}$$

Also, from the second and the fifth equation, we have, respectively,

10 $$\begin{aligned} E= & {} \frac{\beta \rho (1-SD)}{\gamma +\mu } \ SI, \end{aligned}$$

11 $$\begin{aligned} V= & {} \frac{r e^{-\mu \tau }S+\alpha \delta e^{-\mu \tau }I}{\sigma (1-f)I+\phi +\mu }. \end{aligned}$$

Substituting equations (10) and (11) into the third equation in system (9), we get

12 $$\begin{aligned} S=\frac{(\gamma +\mu )\Big [(d+\mu +\delta (1-\alpha e^{-\mu \tau }))\sigma (1-f)I+(\phi +\mu )(\delta +d+\mu )\Big ]}{\gamma \beta \rho (1-SD)(\sigma (1-f)I+\phi +\mu )+\sigma (1-f)(\gamma +\mu )r e^{-\mu \tau }}. \end{aligned}$$

Thus, rewriting (11) and (12), we have

$$\begin{aligned} V= & {} \frac{\gamma \beta \rho (1-SD)\alpha \delta e^{-\mu \tau }I+(\gamma +\mu )(\delta +d+\mu )r e^{-\mu \tau }}{\gamma \beta \rho (1-SD)(\sigma (1-f)I+\phi +\mu )+\sigma (1-f)(\gamma +\mu )r e^{-\mu \tau }}. \\ E= & {} \frac{\beta \rho (1-SD)\Big [(d+\mu +\delta (1-\alpha e^{-\mu \tau }))\sigma (1-f)I^{2}+(\phi +\mu )(\delta +d+\mu )I\Big ]}{\gamma \beta \rho (1-SD)(\sigma (1-f)I+\phi +\mu )+\sigma (1-f)(\gamma +\mu )r e^{-\mu \tau }} . \end{aligned}$$

Finally, substituting *S* and *V* into the first equation in system (9) and solving for *I*, we get the following quadratic equation

13 $$\begin{aligned} a_{2}I^{2}+a_{1}I+a_{0}=0, \end{aligned}$$

where,

$$\begin{aligned} a_{2}&= (\gamma +\mu )(d+\mu +\delta (1-\alpha e^{-\mu \tau }))\beta \rho (1-SD)\sigma (1-f), \\ a_{1}&= (\gamma +\mu )(\phi +\mu )(\delta +d+\mu )\beta \rho (1-SD)-\eta \gamma \beta \rho (1-SD)\sigma (1-f)\\&\quad +(\gamma +\mu )(d+\mu +\delta (1-\alpha e^{-\mu \tau }))(r e^{-\mu \tau }+\mu )\sigma (1-f)-\phi \gamma \beta \rho (1-SD)\alpha \delta e^{-\mu \tau },\\ a_{0}&= \mu (\gamma +\mu )(\delta +d+\mu )(r e^{-\mu \tau }+\phi +\mu )(1-{\mathcal {R}}_c). \end{aligned}$$

Here,

$$\begin{aligned} {\mathcal {R}}_c= \frac{\gamma \eta \beta \rho (1-SD)(\phi +\mu )}{\mu (\gamma +\mu )(\delta +d+\mu )(r e^{-\mu \tau }+\phi +\mu )}+\frac{\eta \sigma (1-f)r e^{-\mu \tau }}{\mu (\delta +d+\mu )(r e^{-\mu \tau }+\phi +\mu )}. \end{aligned}$$

Roots of (13) have the form

$$\begin{aligned} I_{1,2}=\frac{-a_{1}\pm \sqrt{a_{1}^{2}-4a_{2}a_{0}}}{2a_{2}}. \end{aligned}$$

We focus here on studying the existence of the endemic equilibrium when ${\mathcal {R}}_c>1$. Clearly, $a_2>0$ and $a_0<0$. Since $-4a_{2}a_{0}>0$, then $a_{1}\le \sqrt{a_{1}^{2}-4a_{2}a_{0}}$. Hence, if ${\mathcal {R}}_c>1,$ system (7) has a unique endemic equilibrium, $P_{1}=(S_1,E_1,I_1,R_1,V_1)$, where

$$\begin{aligned} S_{1}&= \frac{(\gamma +\mu )\Big [(d+\mu +\delta (1-\alpha e^{-\mu \tau }))\sigma (1-f)I_{1}+(\phi +\mu )(\delta +d+\mu )\Big ]}{\gamma \beta \rho (1-SD)(\sigma (1-f)I_{1}+\phi +\mu )+\sigma (1-f)(\gamma +\mu )r e^{-\mu \tau }}, \\ E_{1}&= \frac{\beta \rho (1-SD)\Big [(d+\mu +\delta (1-\alpha e^{-\mu \tau }))\sigma (1-f)I^{2}_{1}+(\phi +\mu )(\delta +d+\mu )I_{1}\Big ]}{\gamma \beta \rho (1-SD)(\sigma (1-f)I_{1}+\phi +\mu )+\sigma (1-f)(\gamma +\mu )r e^{-\mu \tau }}, \\ I_{1}&= \frac{-a_{1}+ \sqrt{a_{1}^{2}-4a_{2}a_{0}}}{2a_{2}}, \\ R_{1}&= \frac{\delta (1-\alpha e^{-\mu \tau })}{\mu }\ I_{1},\\ V_{1}&= \frac{\gamma \beta \rho (1-SD)\alpha \delta e^{-\mu \tau }I_{1}+(\gamma +\mu )(\delta +d+\mu )r e^{-\mu \tau }}{\gamma \beta \rho (1-SD)(\sigma (1-f)I_{1}+\phi +\mu )+\sigma (1-f)(\gamma +\mu )r e^{-\mu \tau }}. \end{aligned}$$

Therefore, the COVID-19 endemic equilibrium exists only if ${\mathcal {R}}_c>1$.

#### Control reproduction number ${\mathcal {R}}_c$

To find ${\mathcal {R}}_c$, we use the next generation matrix approach^22^. Let ${\mathcal {O}}=(E,I)^T$, then system (7) can be rewritten as $\dot{{\mathcal {O}}} = {\mathscr {F}}({\mathcal {O}}) - {\mathscr {V}} ({\mathcal {O}})$, where

$$\begin{aligned} {\mathscr {F}}= \begin{bmatrix} \beta \rho (1-SD)SI\\ \sigma (1-f)VI \end{bmatrix} \quad \text {and} \quad {\mathscr {V}}= \begin{bmatrix} (\gamma +\mu )E\\ -\gamma E+(\delta +d+\mu )I \end{bmatrix}. \end{aligned}$$

Computing the Jacobian matrix of ${\mathscr {F}}$ and ${\mathscr {V}}$ at $P_{0}$ we obtain, respectively,

$$\begin{aligned} F= \begin{bmatrix} 0 &{} \beta \rho (1-SD)S_{0}\\ 0 &{} \sigma (1-f)V_{0} \end{bmatrix} \quad \text {and} \quad V= \begin{bmatrix} \gamma +\mu &{} 0 \\ -\gamma &{} \delta +d+\mu \end{bmatrix}. \end{aligned}$$

Now, we can evaluate the next generation matrix as follows

$$\begin{aligned} FV^{-1}= \begin{bmatrix} \frac{\gamma \beta \rho (1-SD)S_{0}}{(\gamma +\mu )(\delta +d+\mu )} &{} \frac{\beta \rho (1-SD) S_{0}}{ (\delta +d+\mu )} \\ \frac{\gamma \sigma (1-f)V_{0}}{(\gamma +\mu )(\delta +d+\mu )} &{} \frac{\sigma (1-f)V_{0}}{(\delta +d+\mu )} \end{bmatrix}. \end{aligned}$$

The control reproduction number ${\mathcal {R}}_c$ of system (7) is the spectral radius of matrix $FV^{-1}$, and it is given by

$$\begin{aligned} {\mathcal {R}}_c= \frac{\gamma \eta \beta \rho (1-SD)(\phi +\mu )}{\mu (\gamma +\mu )(\delta +d+\mu )(r e^{-\mu \tau }+\phi +\mu )}+\frac{\eta \sigma (1-f)r e^{-\mu \tau }}{\mu (\delta +d+\mu )(r e^{-\mu \tau }+\phi +\mu )}. \end{aligned}$$

Moreover, we can write the expression of ${\mathcal {R}}_c$ as: ${\mathcal {R}}_{c}=\rho (1-SD){\mathcal {R}}_{S}+(1-f){\mathcal {R}}_{V}$, where ${\mathcal {R}}_S$ and ${\mathcal {R}}_V$ are:

$$\begin{aligned} {\mathcal {R}}_S= & {} \frac{\gamma \eta \beta (\phi +\mu )}{\mu (\gamma +\mu )(\delta +d+\mu )(r e^{-\mu \tau }+\phi +\mu )},\\ {\mathcal {R}}_V= & {} \frac{\eta \sigma r e^{-\mu \tau }}{\mu (\delta +d+\mu )(r e^{-\mu \tau }+\phi +\mu )}. \end{aligned}$$

The interpretation of the terms in ${\mathcal {R}}_c$ are as follows. Firstly, in ${\mathcal {R}}_S$, the term that expresses the incidence of new infections by infected individuals is $\beta SI$. Thus, the number of secondary cases by one infectious individual ($I=1$) in a population containing only susceptible individuals is $\beta S_{0}$, where $S_{0}=\eta (\phi +\mu )/\mu (r e^{-\mu \tau }+\phi +\mu )$. Also, $1/(\delta +d+\mu )$ represents one infectious individual’s average time spent in the infected compartment. In addition, $\gamma /(\gamma +\mu )$ is the proportion of newly infected individuals that survived the incubation period. Secondly, in ${\mathcal {R}}_{V}$, the term that represents the incidence of new infections from the vaccinated class by infected individuals is $\sigma VI$. Then, the number of secondary cases by one infectious individual in a population containing only vaccinated individuals is $\sigma V_{0}$, where $V_{0}=\eta r e^{-\mu \tau }/\mu (r e^{-\mu \tau }+\phi +\mu )$. Again, $1/(\delta +d+\mu )$ represents one infectious individual’s average time spent in the infected compartment.

### Local stability analysis

To investigate the local stability, we compute the Jacobian matrix of system (7),

$$\begin{aligned} J = \begin{bmatrix} J_{11} &{} 0 &{} -\beta \rho (1-SD)S &{} 0 &{} \phi \\ \beta \rho (1-SD)I &{} -(\gamma +\mu ) &{} \beta \rho (1-SD)S &{} 0 &{} 0 \\ 0 &{} \gamma &{} \sigma (1-f)V-(\delta +d+\mu ) &{} 0 &{} \sigma (1-f)I \\ 0 &{} 0 &{} \delta (1-\alpha e^{-\mu \tau }) &{} -\mu &{} 0\\ r e^{-\mu \tau } &{} 0 &{} \delta \alpha e^{-\mu \tau }-\sigma (1-f)V &{} 0 &{} -\sigma (1-f)I-(\phi +\mu ) \end{bmatrix}. \end{aligned}$$

where, $$J_{11}=-\beta \rho (1-SD)I-r e^{-\mu \tau }-\mu$$.

#### COVID-19 free equilibrium point $P_0$

To analyze the local stability of the free equilibrium point $P_0$, we compute the Jacobian matrix of system (7) at $P_0$, which has the form $J_{1}(P_0)+J_{2}(P_0)$, where

$$\begin{aligned} J_{1}(P_0) = \begin{bmatrix} -\mu &{} 0 &{} -\beta \rho (1-SD)S_{0} &{} 0 &{} \phi \\ 0 &{} -(\gamma +\mu ) &{} \beta \rho (1-SD)S_{0} &{} 0 &{} 0 \\ 0 &{} \gamma &{} \sigma (1-f)V_{0}-(\delta +d+\mu ) &{} 0 &{} 0 \\ 0 &{} 0 &{} \delta &{} -\mu &{} 0\\ 0 &{} 0 &{} -\sigma (1-f)V_{0} &{} 0 &{} -(\phi +\mu ) \end{bmatrix}, \end{aligned}$$

and

$$\begin{aligned} J_{2}(P_0) = \begin{bmatrix} -r e^{-\mu \tau } &{} 0 &{} 0 &{} 0 &{} 0 \\ 0 &{} 0 &{} 0 &{} 0 &{} 0 \\ 0 &{} 0 &{} 0 &{} 0 &{} 0 \\ 0 &{} 0 &{} -\alpha \delta e^{-\mu \tau } &{} 0 &{} 0\\ r e^{-\mu \tau } &{} 0 &{} \alpha \delta e^{-\mu \tau } &{} 0 &{} 0 \end{bmatrix}. \end{aligned}$$

Then, solving the characteristic equation $|J_{1}(P_0)+J_{2}(P_0)e^{-\uplambda \tau }+\uplambda I|=0$, we get

14 $$\begin{aligned} \Big (\mu +\uplambda \Big )^2\Big (r e^{-(\uplambda +\mu )\tau }+\phi +\mu +\uplambda \Big )\Big (\uplambda ^2+c_1\uplambda +c_0 \Big )=0, \end{aligned}$$

where

$$\begin{aligned} c_1&=\gamma +\mu +(\delta +d+\mu )\Big (1-(1-f){\mathcal {R}}_v\Big ),\\ c_0&=(\gamma +\mu )(\delta +d+\mu )(1-{\mathcal {R}}_c). \end{aligned}$$

##### Theorem 2

If ${\mathcal {R}}_c<1$, the COVID-19 free equilibrium point $P_0$, is locally asymptotically stable for $\tau \ge \tau ^{*}$, where

$$\begin{aligned} \tau ^{*}=\frac{1}{\mu }\Big (\ln {\frac{r}{\phi +\mu }} \Big ). \end{aligned}$$

##### Proof

The roots of the characteristic equation (14) are $\uplambda _{1,2}=-\mu$ and $\uplambda _{3,4}$ satisfy the following equation

$$\begin{aligned} \uplambda ^2+c_1\uplambda +c_0=0. \end{aligned}$$

Using the Routh-Hurwitz criteria, $\uplambda _{3,4}$ have a negative real part if $c_1>0$ and $c_0>0$. The coefficients $c_1$ and $c_0$ are positive if ${\mathcal {R}}_c=\rho (1-SD){\mathcal {R}}_S+(1-f){\mathcal {R}}_V<1$. Hence, we assume that ${\mathcal {R}}_c<1$. The last root of (14) satisfies

15 $$\begin{aligned} \uplambda +\phi +\mu +r e^{-(\mu +\uplambda )\tau }=0. \end{aligned}$$

When $\tau =0$, then $\uplambda _5=-(\phi +\mu +r)$. Therefore, $P_0$ is locally asymptotically stable for case $\tau =0$. For $\tau >0$, we assume that $\uplambda =i\omega ,\ (\omega \in {\mathbb {R}})$ is a root of (15). Substituting $\uplambda =i\omega$ in (15), we have

$$\begin{aligned} i\omega +\phi +\mu +r e^{-(i\omega +\mu )\tau }=0. \end{aligned}$$

Then by using Euler’s formula $e^{-i\tau \omega }=\cos (\tau \omega )-i \sin (\tau \omega )$, and equating the real and imaginary parts, we obtain

$$\begin{aligned} \phi +\mu&=-r e^{-\mu \tau }\cos (\tau \omega ),\\ \omega&=r e^{-\mu \tau }\sin (\tau \omega ). \end{aligned}$$

Squaring the above equations and adding them, we get

$$\begin{aligned} \omega =\sqrt{\Big (r e^{-\mu \tau }-(\phi +\mu )\Big )\Big (r e^{-\mu \tau }+(\phi +\mu ) \Big )}. \end{aligned}$$

Hence, the purely imaginary root exists if and only if $\omega \in {\mathbb {R}}$. Thus,

$$\begin{aligned} r e^{-\mu \tau }-(\phi +\mu )>0&\Longrightarrow \tau \ <\frac{1}{\mu }\ln {\frac{r}{\phi +\mu }}=\tau ^{*}. \end{aligned}$$

If the delay value $\tau <\tau ^{*}$, then equation (15) has the purely imaginary root. Therefor, the stability of $P_0$ can change from stable to unstable. Conversely, if $\tau >\tau ^{*}$ the point $P_0$ is stable. Hence, if $R_c<1$ the COVID-19 free equilibrium is locally asymptotically stable when $\tau \ge \tau ^{*}$.

#### COVID-19 endemic equilibrium point $P_1$

We study the local stability of the endemic equilibrium point, $P_1$, assuming it exists, by evaluating the Jacobian matrix of system (7) around $P_1$, we obtain

$$\begin{aligned} J(P_{1}) = \begin{bmatrix} J_{11} &{} 0 &{} -\beta \rho (1-SD)S_1 &{} 0 &{} \phi \\ \beta \rho (1-SD)I_1 &{} -(\gamma +\mu ) &{} \beta \rho (1-SD)S_1 &{} 0 &{} 0 \\ 0 &{} \gamma &{} \sigma (1-f)V_1-(\delta +d+\mu ) &{} 0 &{} \sigma (1-f)I_1 \\ 0 &{} 0 &{} \delta (1-\alpha e^{-\mu \tau }) &{} -\mu &{} 0\\ r e^{-\mu \tau } &{} 0 &{} \delta \alpha e^{-\mu \tau }-\sigma (1-f)V_1 &{} 0 &{} -\sigma (1-f)I_1-(\phi +\mu ) \end{bmatrix}, \end{aligned}$$

where, $J_{11}=-\beta \rho (1-SD)I_1-r e^{-\mu \tau }-\mu$. From the equilibrium equation (9) of $P_1$, we have $-\big [\beta \rho (1-SD)I_1+r e^{-\mu \tau }+\mu \big ]=-(\eta +\phi V_1)/S_1$. The matrix $J(P_1)$ can be written in the form, $J_{1}(P_1)+J_{2}(P_1)$, where

$$\begin{aligned} J_{1}(P_1) = \begin{bmatrix} \frac{-(\eta +\phi V_1)}{S_1} &{} 0 &{} -\beta \rho (1-SD)S_{1} &{} 0 &{} \phi \\ \beta \rho (1-SD)I_{1} &{} -(\gamma +\mu ) &{} \beta \rho (1-SD)S_{1} &{} 0 &{} 0 \\ 0 &{} \gamma &{} J_{33} &{} 0 &{} \sigma (1-f)I_{1} \\ 0 &{} 0 &{} \delta &{} -\mu &{} 0\\ 0 &{} 0 &{} -\sigma (1-f)V_{1} &{} 0 &{} J_{55} \end{bmatrix}, \end{aligned}$$

and

$$\begin{aligned} J_{2}(P_1) = \begin{bmatrix} 0 &{} 0 &{} 0 &{} 0 &{} 0 \\ 0 &{} 0 &{} 0 &{} 0 &{} 0 \\ 0 &{} 0 &{} 0 &{} 0 &{} 0 \\ 0 &{} 0 &{} -\alpha \delta e^{-\mu \tau } &{} 0 &{} 0\\ r e^{-\mu \tau } &{} 0 &{} \alpha \delta e^{-\mu \tau } &{} 0 &{} 0 \end{bmatrix}, \end{aligned}$$

where, $J_{33}=\sigma (1-f)V_{1}-(\delta +d+\mu )$, and $J_{55}=-\sigma (1-f)I_{1}-(\phi +\mu )$. The characteristic equation of the linearized system (7) at $P_{1}$ is in the form

16 $$\begin{aligned} (\uplambda +\mu )\Big [{\mathcal {P}}_{1}(\uplambda )+{\mathcal {P}}_{2}(\uplambda ,\tau )e^{-\uplambda \tau } \Big ]=0, \end{aligned}$$

where

$$\begin{aligned} {\mathcal {P}}_{1}&= \uplambda ^{4}+A_{3}\uplambda ^{3}+A_{2}\uplambda ^{2}+A_{1}\uplambda +A_{0}, \\ {\mathcal {P}}_{2}&= e^{-\mu \tau }\Big (B_{2}\uplambda ^{2}+B_{1}\uplambda +B_{0}\Big ), \end{aligned}$$

where

$$\begin{aligned} A_{3}&=\Big (\frac{\eta +\phi V_1}{S_1}\Big )+\sigma (1-f)(I_{1}-V_{1})+\delta +\gamma +d+\phi +3\mu ,\\ A_{2}&=\Big (\delta +d+\mu \Big )\Big (\sigma (1-f)I_{1}+\phi +\mu \Big )-(\phi +\mu )\sigma (1-f)V_{1} \\ \quad&\quad +\Big (\frac{\eta +\phi V_1}{S_1}+\gamma +\mu \Big )\Big (\sigma (1-f)(I_{1}-V_{1})+\phi +\delta +d+2\mu \Big )\\ \quad&\quad +\Big (\frac{\eta +\phi V_1}{S_1}\Big )\Big (\gamma +\mu \Big )-\gamma \beta \rho (1-SD)S_{1}, \\ A_{1}&=\Big (\frac{\eta +\phi V_1}{S_1}\Big )\Big (\gamma +\mu \Big )\Big (\sigma (1-f)(I_{1}-V_{1})+\delta +d+\phi +2\mu \Big )\\ \quad&\quad +\Big (\frac{\eta +\phi V_1}{S_1}+\gamma +\mu \Big )\Big (\delta +d+\mu \Big )\Big (\sigma (1-f)I_{1}+\phi +\mu \Big ) \\ \quad&\quad -\Big (\frac{\eta +\phi V_1}{S_1}+\gamma +\mu \Big )\Big ((\phi +\mu )\sigma (1-f)V_{1} \Big )+\Big (\beta \rho (1-SD)\Big )^{2}S_{1}I_{1} \\ \quad&\quad -\gamma \beta \rho (1-SD)\Big (\eta +\phi V_{1}\Big )-\gamma \beta \rho (1-SD)S_{1}\Big (\sigma (1-f)I_{1}+\phi +\mu \Big ),\\ A_{0}&=-\gamma \beta \rho (1-SD)\Big (\sigma (1-f)I_{1}+\phi +\mu \Big )\Big (\beta \rho (1-SD)S_{1}I_{1}-(\eta +\phi V_{1}) \Big )\\ \quad&\quad -\Big (\frac{\eta +\phi V_1}{S_1}\Big )\Big (\gamma +\mu \Big )\Big ((\phi +\mu )\sigma (1-f)V_{1}\Big )+\phi \gamma \beta \rho (1-SD)\sigma (1-f)I_{1}V_{1} \\ \quad&\quad +\Big (\frac{\eta +\phi V_1}{S_1}\Big )\Big (\gamma +\mu \Big )\Big (\delta +d+\mu \Big )\Big (\sigma (1-f)I_{1}+\phi +\mu \Big ),\\ B_{2}&= -\phi r-\alpha \delta \sigma (1-f)I_{1},\\ B_{1}&= r\Big (\beta \rho (1-SD)\sigma (1-f)S_{1}I_{1}+\phi \sigma (1-f)V_{1}-\phi (\delta +d+\mu ) \Big )\\ \quad&\quad -\Big (\frac{\eta +\phi V_{1}}{S_{1}}+\gamma +\mu \Big ) \alpha \delta \sigma (1-f) I_{1}-\phi r(\gamma +\mu ),\\ B_{0}&=r\Big (\gamma +\mu \Big )\Big (\beta \rho (1-SD)\sigma (1-f)S_{1}I_{1}+\phi \sigma (1-f)V_{1}-\phi (\delta +d+\mu ) \Big )\\ \quad&\quad -\phi \gamma \beta \rho (1-SD)\Big (\alpha \delta I_{1}-r S_{1}\Big )-\Big (\frac{\eta +\phi V_{1}}{S_{1}}\Big )\Big (\gamma +\mu \Big ) \alpha \delta \sigma (1-f) I_{1}. \end{aligned}$$

##### Theorem 3

At the absence of a delay, that is, $\tau =0$, the COVID-19 endemic equilibrium $P_1$, if it exists, is locally asymptotically stable if and only if the following conditions hold:

17 $$\begin{aligned} \begin{aligned} {\mathcal {K}}_{1}>0, \quad {\mathcal {K}}_{2}>0, \quad {\mathcal {K}}_{3}>0, \quad&{\mathcal {K}}_{4}>0,\\ {\mathcal {K}}_{1}{\mathcal {K}}_{2}{\mathcal {K}}_{3}>{\mathcal {K}}_{3}^{2}+{\mathcal {K}}_{1}^{2}{\mathcal {K}}_{4}, \end{aligned} \end{aligned}$$

where

$$\begin{aligned} \begin{aligned} {\mathcal {K}}_{1}&=A_{3}, \quad&{\mathcal {K}}_{3}=A_{1}+B_{1}, \\ {\mathcal {K}}_{2}&=A_{2}+B_{2}, \quad&{\mathcal {K}}_{4}=A_{0}+B_{0}. \end{aligned} \end{aligned}$$

##### Proof

When $\tau =0$, the characteristic equation (16) has $\uplambda _{1}=-\mu$ and the other roots are the solution to the following equation

$$\begin{aligned} {\mathcal {P}}_{1}(\uplambda )+{\mathcal {P}}_{2}(\uplambda )=0, \end{aligned}$$

that is,

18 $$\begin{aligned} \uplambda ^{4}+A_3\uplambda ^{3}+(A_2+B_2)\uplambda ^{2}+(A_1+B_1)\uplambda +(A_0+B_0)=0. \end{aligned}$$

Therefore, using the Routh-Hurwitz criteria, if the conditions in (17) are satisfied, then the roots of equation (18) are either negative or have a negative real part. Hence, if $P_1$ exists, it is locally asymptotically stable without delay.

To analyze the second case, when $\tau >0$, the characteristic equation (16) has $\uplambda _{1}=-\mu$. Assuming that $\uplambda =i\omega ,\ (\omega \in {\mathbb {R}})$ is a root of (16), we get

19 $$\begin{aligned} {\mathcal {P}}_{1}(i\omega )+{\mathcal {P}}_{2}(i\omega ,\tau )e^{-i\omega \tau }=0. \end{aligned}$$

Separating the real and imaginary parts of (19), we obtain respectively,

20 $$\begin{aligned}{}&R_{1}(\omega )+R_{2}(\omega )\cos (\tau \omega )+Q_{2}(\omega )\sin (\tau \omega )=0 \end{aligned}$$

21 $$\begin{aligned}{}&Q_{1}(\omega )+Q_{2}(\omega )\cos (\tau \omega )-R_{2}(\omega )\sin (\tau \omega )=0 \end{aligned}$$

where

$$\begin{aligned} \begin{aligned} R_{1}&=\omega ^4-A_2\omega ^2+A_0, \quad&Q_{1}&=-A_3\omega ^3+A_1\omega , \\ R_{2}&=e^{-\mu \tau }\Big (-B_2\omega ^2+B_0\Big ), \quad&Q_{2}&=e^{-\mu \tau }B_1\omega . \end{aligned} \end{aligned}$$

Squaring (20) and (21), and adding them we obtain

$$\begin{aligned} \begin{aligned} \omega ^8+{\mathcal {G}}_{3}\omega ^6+{\mathcal {G}}_{2}\omega ^4+{\mathcal {G}}_{1}\omega ^2+{\mathcal {G}}_{0}=0, \end{aligned} \end{aligned}$$

where

$$\begin{aligned} {\mathcal {G}}_{3}&=A_{3}^{2}-2A_{2},\\ {\mathcal {G}}_{2}&=A_{2}^{2}+2A_{0}-2A_{1}A_{3}-e^{-2\mu \tau }B_{2}^{2},\\ {\mathcal {G}}_{1}&=A_{1}^{2}-2A_{0}A_{2}+e^{-2\mu \tau }\Big (2B_{0}B_{2}-B_{1}^{2} \Big ),\\ {\mathcal {G}}_{0}&=A_{0}^{2}-e^{-2\mu \tau }B_{0}^{2}. \end{aligned}$$

Let $$Z=\omega ^{2}$$, then

22 $$\begin{aligned} \begin{aligned} Z^4+{\mathcal {G}}_{3}Z^3+{\mathcal {G}}_{2}Z^2+{\mathcal {G}}_{1}Z+{\mathcal {G}}_{0}=0. \end{aligned} \end{aligned}$$

When the coefficients of (22) satisfy the Routh-Hurwitz criterion, that is,

23 $$\begin{aligned} \begin{aligned} {\mathcal {G}}_{3}>0, \quad {\mathcal {G}}_{2}>0, \quad {\mathcal {G}}_{1}>0, \quad&{\mathcal {G}}_{0}>0,\\ {\mathcal {G}}_{1}{\mathcal {G}}_{2}{\mathcal {G}}_{3}>{\mathcal {G}}_{1}^{2}+{\mathcal {G}}_{3}^{2}{\mathcal {G}}_{0}, \end{aligned} \end{aligned}$$

then all roots of (22) are negative or have negative real parts. Accordingly, $\omega =\sqrt{Z}$, however, this contradicts our assumption that $\omega \in {\mathbb {R}}$. Thus, the signs of the eigenvalues remain the same as in case $\tau =0$ and do not change as $\tau$ increases. The following theorems summarize the stability of $P_1$ when $\tau >0$.

##### Theorem 4

If the roots of (22) satisfy the conditions (23), then the COVID-19 endemic equilibrium $P_1$ is locally asymptotically stable for $\tau >0$, provided it is stable at $\tau =0$.

##### Theorem 5

When the COVID-19 endemic equilibrium is stable at $\tau =0$, its stability will change to unstable when there is a delay if (22) has at least one positive root, meaning that the stability conditions (23) are not met.

## Numerical analysis

In this section, we perform numerical simulations for system (7) to show the agreement with the qualitative analysis results and examine the effect of some parameters. In addition, we analyze the sensitivity of the system’s parameters for the threshold quantity, the control reproduction number, ${\mathcal {R}}_c$. Table 1 shows the values for the system’s parameters that mainly were used in the numerical analysis of this model.

**Table 1:** The description and values of the parameters in model (7).

Parameter	Description	Value	Unit	Source
$\eta$	Birth rate	1250	Individual $\times$ Day$^{-1}$	^23^
$\mu$	Natural death rate	0.04	Day$^{-1}$	Estimated
$\beta$	Transmission rate	1.0063 $\times$ 10$^{-7}$	(Individual $\times$ Day)$^{-1}$	^23^
$\gamma$	Incubation rate	0.167	Day$^{-1}$	^24^
$\delta$	Recovery rate	$3.2772\times 10^{-1}$	Day$^{-1}$	^23^
*d*	Death rate due to COVID-19	$2.3724\times 10^{-1}$	Day$^{-1}$	^23^
$\rho$	Lockdown	0.85	–	Estimated
*SD*	Social distancing	0.3	–	Estimated
$\tau$	Time delay	120	Day	Estimated
*r*	Vaccination rate	0.6	Day$^{-1}$	Estimated
*f*	Efficiency of vaccine	0.4	–	Estimated
$\sigma$	Infection after vaccine rate	0.00055	(Individual $\times$ Day)$^{-1}$	Estimated
$\phi$	Loss of vaccine immunity rate	0.0005	Day$^{-1}$	Estimated
$\alpha$	Percentage of *R* receive the vaccine	0.7	–	Estimated

### Numerical experiments

We re-scale the state variables in the system before implementing the numerical analysis. Let

24 $$\begin{aligned} S={\widehat{S}}N,\ E={\widehat{E}}N,\ I={\widehat{I}}N,\ R={\widehat{R}}N,\ V={\widehat{V}}N. \end{aligned}$$

Substituting (24) in system (7) and using the limiting value of *N* as *t* increases, that is, $N=\eta /\mu$, we obtain the following re-scaled system (omitting the hat onward)

25 $$\begin{aligned} \begin{aligned} \frac{dS}{dt}&=\mu -\beta \rho (1-SD)\frac{\eta }{\mu }SI-r S(t-\tau )e^{-\mu \tau }+\phi V-\mu S, \\ \frac{dE}{dt}&=\beta \rho (1-SD)\frac{\eta }{\mu }SI-(\gamma +\mu )E,\\ \frac{dI}{dt}&=\gamma E+\sigma (1-f)\frac{\eta }{\mu } VI-(\delta +d+\mu )I,\\ \frac{dR}{dt}&=\delta I-\alpha \delta I(t-\tau )e^{-\mu \tau }-\mu R,\\ \frac{dV}{dt}&=r S(t-\tau )e^{-\mu \tau }+\alpha \delta I(t-\tau ) e^{-\mu \tau }-\sigma (1-f)\frac{\eta }{\mu } VI-(\phi +\mu )V. \end{aligned} \end{aligned}$$

The system (25) is solved numerically using MATLAB package dde23, a package to solve delay systems with constant delays. We set the value zero to the parameters related to the vaccine during the delay interval, i.e., before producing the vaccine, that is, $r=\phi =\sigma =f=\alpha =0$. Accordingly, we used the conditional statements when defining system (25) in the MATLAB function file to set two different values to the parameters before and after the production of the vaccine (see Supplementary Material).

The first two experiments examines the stability of the equilibrium points, therefore, numerical simulations are executed for three different initial histories, where $\zeta \in [-\tau ,0]$:

$$\begin{aligned}&IH1: S(\zeta )=0.8,\ E(\zeta )=0.1,\ I(\zeta )=0.05,\ R(\zeta )=0,\ V(\zeta )=0,\\&IH2: S(\zeta )=0.6,\ E(\zeta )=0.2,\ I(\zeta )=0.15,\ R(\zeta )=0,\ V(\zeta )=0,\\&IH3: S(\zeta )=0.4,\ E(\zeta )=0.3,\ I(\zeta )=0.20,\ R(\zeta )=0,\ V(\zeta )=0. \end{aligned}$$

**Experiment 1:** **Stability of COVID-19 free equilibrium.** We consider the parameters of system (25) as in Table 1 with some modification as follows: $\rho =0.5,\ SD=0.75,\ r=0.9,\ \phi =0.0001,\ \sigma =0.0001,\ f=0.9,$ and $\alpha =0.0001.$ Then, the critical value of the delay will be $\tau ^{*}=77.7755$ (see Theorem 2). Therefore, we examine two cases for the time delay $\tau$.

**Figure 2:**
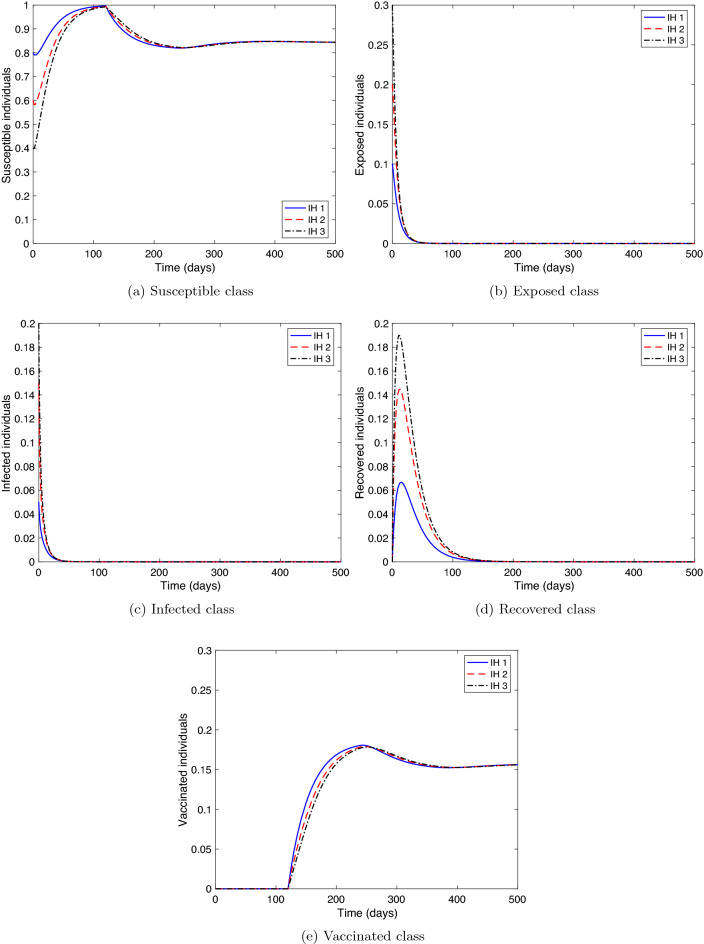
Numerical solution of system (25) versus time with different initial histories for ${\mathcal {R}}_{c}<1$ and $\tau \ge \tau ^{*}$.

**Figure 3:**
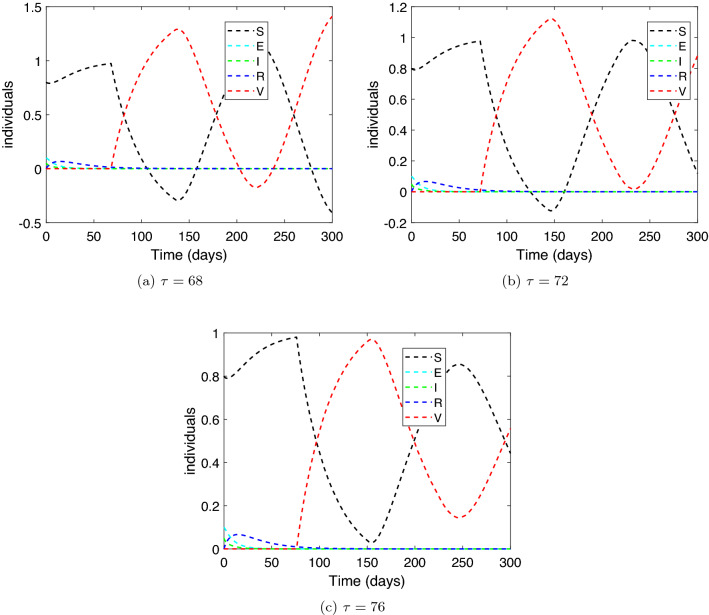
The numerical solution of system (25) versus time for ${\mathcal {R}}_{c}<1$ and $\tau <\tau ^{*}$.

(i) Case $\tau >\tau ^{*}$. Let $\tau =120$, then, ${\mathcal {R}}_c=0.5381<1$ and the equilibrium value is $P_0=(0.8441,0,0,0,0.1559)$. Figure 2 displays the solution curves of the model where they tend to the free equilibrium point, $P_0$, with different initial histories.
(ii) Case $\tau <\tau ^{*}$. Set different values for $\tau$ as: $\tau =68,\ 72,\ \text {and}\ 76$. Figure 3 shows that the free equilibrium point, $P_0$, lost its stability state. A periodic solution appears due to the presence of a pair of purely imaginary roots for the linearized system of (25) around $P_0$. Therefore, Hopf bifurcation occurs.

Hence, numerical simulations of Experiment 1 confirm the qualitative results in Theorem 2.

**Figure 4:**
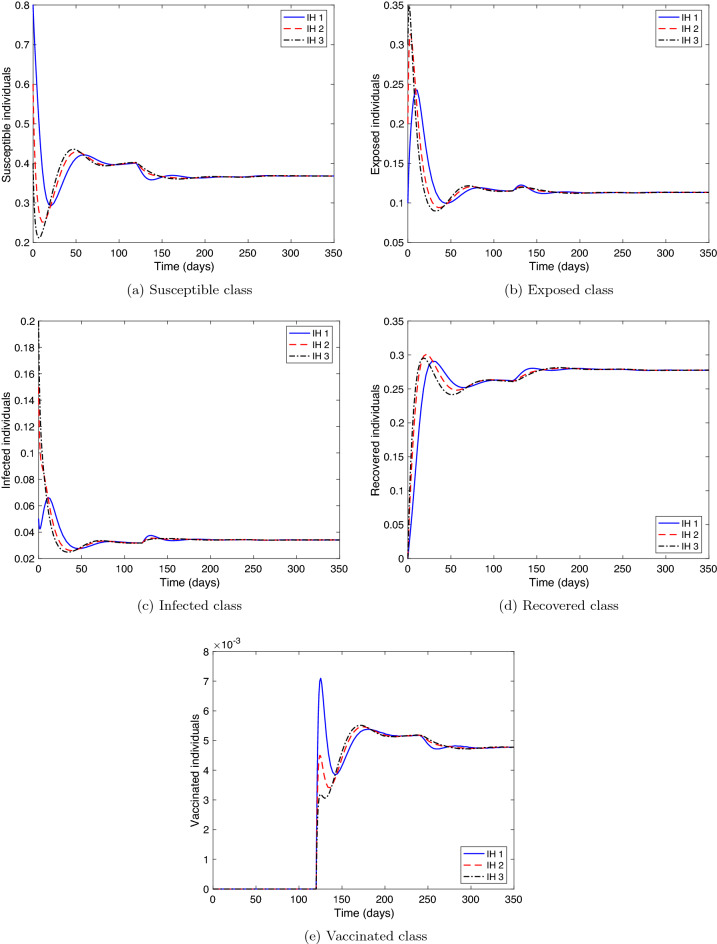
Numerical solution of system (25) versus time with different initial histories where ${\mathcal {R}}_{c}>1$, and stability conditions (17) and (23) are satisfied.

**Figure 5:**
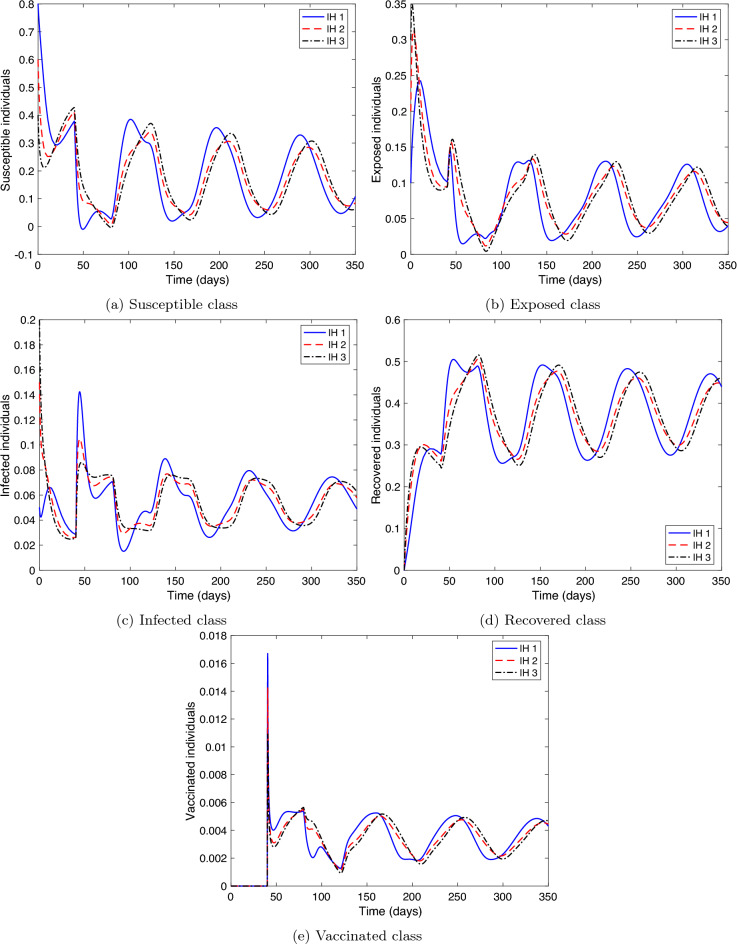
Numerical solution of system (25) versus time with different initial histories where ${\mathcal {R}}_{c}>1$, and the stability conditions (17) are satisfied, but conditions in (23) are not satisfied.

**Experiment 2:** **Stability of COVID-19 endemic equilibrium.** We consider two cases for the values of the parameters of system (25) as follows:

(i) Let the parameters as in Table 1. Therefore, ${\mathcal {R}}_c= 4.0766>1$ and these values fulfill the stability conditions (17) at $\tau =0$ and the conditions (23) when $\tau >0$. Figure 4 presents the solution curves arriving at the endemic equilibrium point, $P_1=(0.3680,\ 0.1134,\ 0.0341,\ 0.2776,\ 0.0048)$. Hence, the result of this experiment is consistent with the result of Theorem 4.
(ii) Let $\tau =40$. For the vaccine parameters, we choose their values after vaccine production to be: $\ r=0.5,\ \phi =0.005,\ \sigma =0.0055,\ f=0.4,\ \text {and}\ \alpha =0.7.$ Then, ${\mathcal {R}}_c=118.6757>1$. These values satisfy the stability conditions (17) at $\tau =0$. However, ${\mathcal {G}}_{1}<0$ and ${\mathcal {G}}_{1}{\mathcal {G}}_{2}{\mathcal {G}}_{3}-({\mathcal {G}}_{1}^{2}+{\mathcal {G}}_{3}^{2}{\mathcal {G}}_{0})<0$, which means that the stability conditions (23) are not met. Figure 5 coincides with Theorem 5 where it illustrates the instability of the endemic equilibrium point $P_1$.

In the remaining experiments, we consider the first initial history (IH1) in the analysis.

**Figure 6:**
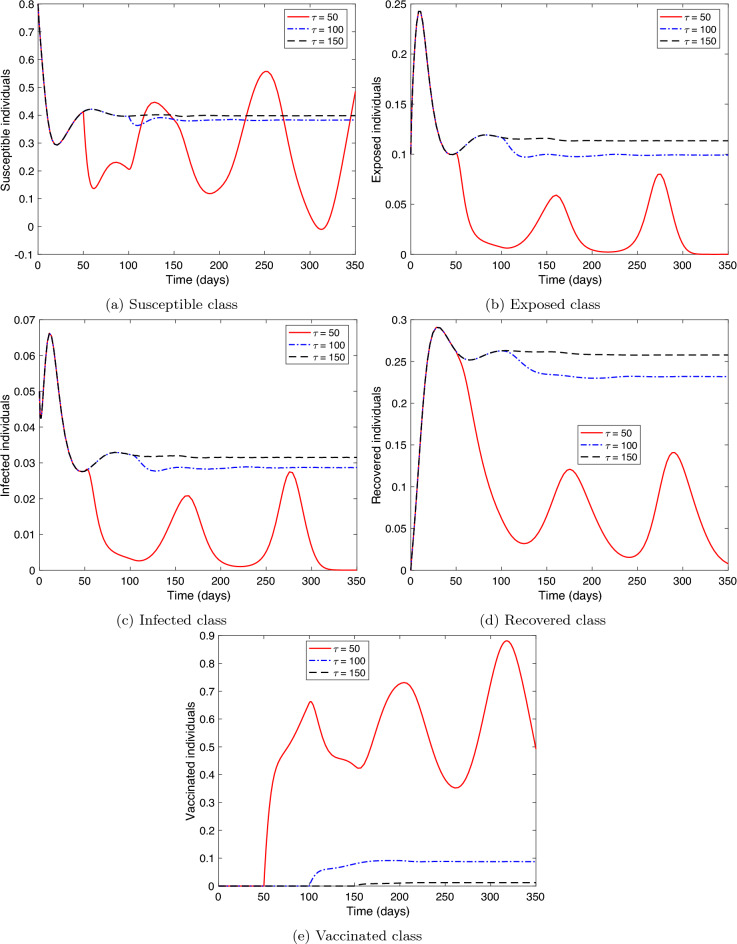
Numerical solution of system (25) versus time with different values for the time delay $\tau$.

**Experiment 3:** **Effect of the time delay on the system dynamics.** We examine different time delay values, $\tau =50,\ 100$, and 150. Let $\sigma =0.0001,\ f=0.9,$ where the rest of the parameters are as in Table 1. Figure 6 displays that as $\tau$ decreases, the model’s compartments oscillate and illustrate a behavior similar to the instability of the endemic equilibrium point, $P_1$. However, when $\tau$ increases, the compartments reach a stable equilibrium level. This is consistent with the result of the sensitivity analysis of ${\mathcal {R}}_c$ concerning $\tau$ in the next subsection (see Fig. 9); ${\mathcal {R}}_c$ increases with an increase in $\tau$ to a certain extent, and then reaches an equilibrium limit that is not affected by the rise in $\tau$. Note that although instability occurs when $\tau$ is less than $\tau ^{*}$, the size of infected individuals decreases since as $\tau$ increases, ${\mathcal {R}}_c$ exceeds the value one.

**Figure 7:**
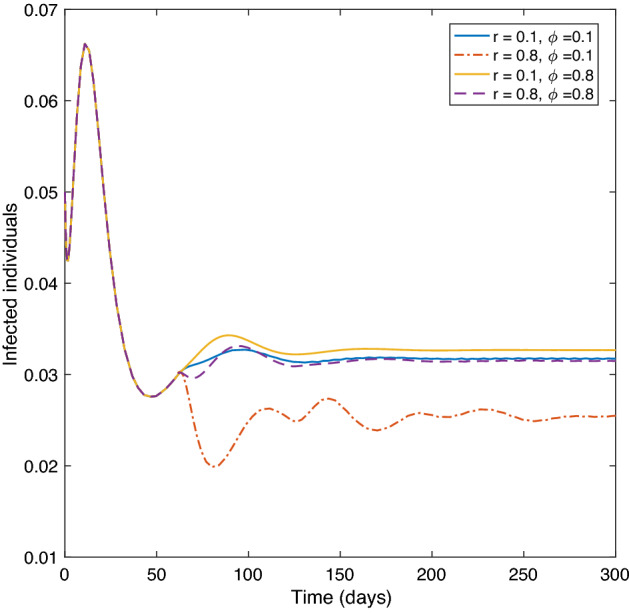
The effect of *r* and $\phi$ on the infected class of system (25).

**Experiment 4:Effect of the vaccination rate and loss of immunity vaccine rate on infected class.** Let $\sigma =0.0001,\ f=0.9,$ and $\tau =60$, where the remaining parameters are as in Table 1. We analyze the impact on the infected class when varying the vaccination rate, *r*, with the loss of immunity rate, $\phi$. Figure 7 shows that when both rates take low values, $r=\phi =0.1$, or high values $r=\phi =0.8$ the size of the infected class approaches a value near the equilibrium value. However, when the vaccination rate is high, $r=0.8,$ with a low loss of immunity rate, $\phi =0.1,$ the equilibrium level of the infected class drops down. Meanwhile, if the vaccination rate is low, $r=0.1$ with a high loss of immunity rate, $\phi =0.8$, the size of the infected class rises to a higher level than the equilibrium value. This indicates the importance of increasing the vaccination rate while decreasing the loss of immunity rate.

**Figure 8:**
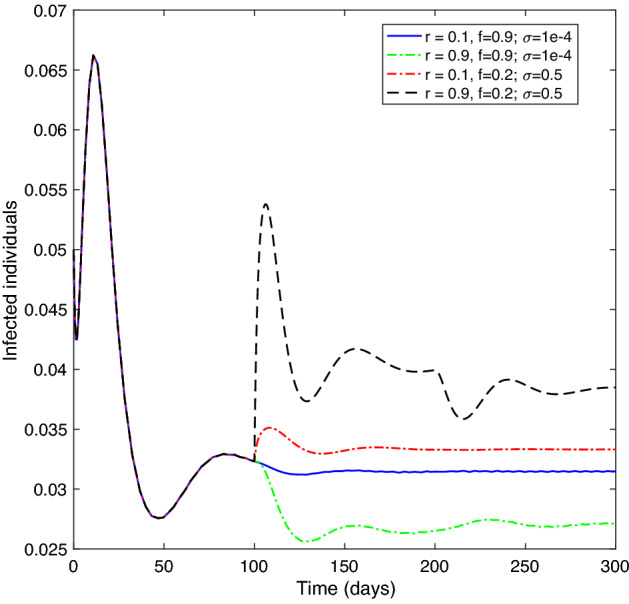
The effect of $r,\ f$ and $\sigma$ on the infected class of system (25).

**Experiment 5:** **Effect of the vaccination rate and the vaccine efficiency on infected class.** Let $\tau =100$, where the remaining parameters are as in Table 1. Figure 8 displays that when the vaccination rate is low, $r=0.1,$ a high-efficiency vaccine ratio, $f=0.9\ \text {and}\ \sigma =0.0001,$ the infected class size tends to be near the equilibrium level. However, when the efficiency ratio of the vaccine is low, $f=0.2,\ \text {and}\ \sigma =0.5,$ with a low vaccination rate, $r=0.1$, the size of the infected class rises above the equilibrium level. Meanwhile, if the vaccination rate is high, $r=0.9$; the size of the infected class drops below the equilibrium level when the vaccine has a high ratio of efficiency, $f=0.9,\ \text {and}\ \sigma =0.0001$; and rises above the equilibrium when the ratio of vaccine efficiency is low, $f=0.2,\ \text {and} \ \sigma =0.5.$

This implies that even if the vaccination rate is high, the infected class’s size drops only if the vaccine has a high-efficiency ratio.

### Sensitivity analysis for ${\mathcal {R}}_{c}$

To know the effect of the parameters in system (7) on the control reproduction number ${\mathcal {R}}_c$, we perform the sensitivity analysis on ${\mathcal {R}}_c$. This analysis is investigated analytically through computing $\partial {\mathcal {R}}_c/\partial {\mathcal {P}}$, where, ${\mathcal {P}}=(\eta ,\ \beta ,\ \rho ,\ SD,\ r,\ \phi ,\ \gamma ,\ \sigma ,\ \delta ,\ d,\ \mu )$. Note that, ${\mathcal {R}}_c$ for system (7) does not depend on $\alpha$. The sensitivity of ${\mathcal {R}}_c$ to each parameter is as follows:

26 $$\begin{aligned} \begin{aligned} \frac{\partial {\mathcal {R}}_c}{\partial \sigma }&=\frac{\eta r e^{-\mu \tau }(1-f)}{\mu (\delta +d+\mu )(r e^{-\mu \tau }+\phi +\mu )}>0,\\ \frac{\partial {\mathcal {R}}_c}{\partial f}&=\frac{-r e^{-\mu \tau }}{\mu (\delta +d+\mu )(r e^{-\mu \tau }+\phi +\mu )}<0,\\ \frac{\partial {\mathcal {R}}_c}{\partial \tau }&=\frac{\eta \sigma r e^{-\mu \tau }(\phi +\mu )(1-f)}{(\delta +d+\mu )(r e^{-\mu \tau }+\phi +\mu )^2}(\Psi -1),\\ \frac{\partial {\mathcal {R}}_c}{\partial \phi }&=\frac{\eta r e^{-\mu \tau }\sigma (1-f)}{\mu (\delta +d+\mu )(r e^{-\mu \tau }+\phi +\mu )^2}(\Psi -1),\\ \frac{\partial {\mathcal {R}}_c}{\partial r}&=\frac{\eta \sigma e^{-\mu \tau }(\phi +\mu )(1-f)}{\mu (\delta +d+\mu )(r e^{-\mu \tau }+\phi +\mu )^2}(1-\Psi ),\\ \frac{\partial {\mathcal {R}}_c}{\partial \beta }&=\frac{\eta \gamma \rho (1-SD)(\phi +\mu )}{\mu (\gamma +\mu )(\delta +d+\mu )(r e^{-\mu \tau }+\phi +\mu )}>0,\\ \frac{\partial {\mathcal {R}}_c}{\partial \rho }&=\frac{\eta \gamma \beta (1-SD)(\phi +\mu )}{\mu (\gamma +\mu )(\delta +d+\mu )(r e^{-\mu \tau }+\phi +\mu )}>0,\\ \frac{\partial {\mathcal {R}}_c}{\partial SD}&=\frac{-\eta \gamma \beta \rho (\phi +\mu )}{\mu (\gamma +\mu )(\delta +d+\mu )(r e^{-\mu \tau }+\phi +\mu )}<0,\\ \frac{\partial {\mathcal {R}}_c}{\partial \gamma }&=\frac{\eta \beta \rho (1-SD)(\phi +\mu )}{(\gamma +\mu )^2(\delta +d+\mu )(r e^{-\mu \tau }+\phi +\mu )}>0,\\ \frac{\partial {\mathcal {R}}_c}{\partial \delta }&=\frac{-\eta \Big [\gamma \beta \rho (1-SD)(\phi +\mu )+r e^{-\mu \tau }\sigma (1-f)(\gamma +\mu ) \Big ]}{\mu (\gamma +\mu )(\delta +d+\mu )^2(r e^{-\mu \tau }+\phi +\mu )}<0,\\ \frac{\partial {\mathcal {R}}_c}{\partial d}&=\frac{-\eta \Big [\gamma \beta \rho (1-SD)(\phi +\mu )+r e^{-\mu \tau }\sigma (1-f)(\gamma +\mu ) \Big ]}{\mu (\gamma +\mu )(\delta +d+\mu )^2(r e^{-\mu \tau }+\phi +\mu )}<0,\\ \frac{\partial {\mathcal {R}}_c}{\partial \eta }&=\frac{\gamma \beta \rho (1-SD)(\phi +\mu )}{\mu (\gamma +\mu )(\delta +d+\mu )(r e^{-\mu \tau }+\phi +\mu )}+\frac{r e^{-\mu \tau } \sigma (1-f)}{\mu (\delta +d+\mu )(r e^{-\mu \tau }+\phi +\mu )}>0,\\ \frac{\partial {\mathcal {R}}_c}{\partial \mu }&=\frac{-\eta \Big [\gamma \beta \rho (1-SD)\Theta _{1}+r e^{-\mu \tau } \sigma (1-f)(\gamma +\mu )^2\Theta _{2} \Big ]}{\mu ^2(\gamma +\mu )^2(\delta +d+\mu )^2(r e^{-\mu \tau }+\phi +\mu )^2}<0, \end{aligned} \end{aligned}$$

where,

$$\begin{aligned} \Theta _{1}&= \mu (\phi +\mu )\Big [(\gamma +\mu )(\delta +d+\mu )(1-r e^{-\mu \tau } \tau )+(r e^{-\mu \tau }+\phi +\mu )(\delta +d+\gamma +2\mu ) \Big ] \\&\quad +\phi (\gamma +\mu )(\delta +d+\mu )(r e^{-\mu \tau }+\phi +\mu ),\\ \Theta _{2}&= (\delta +d+\mu )\Big [r e^{-\mu \tau }+\tau \mu (\phi +\mu )+\phi +\mu +1 \Big ]+\mu (r e^{-\mu \tau }+\phi +\mu ),\\ \Psi&=\frac{\gamma \beta \rho (1-SD)}{(\gamma +\mu )\sigma (1-f)}. \end{aligned}$$

**Figure 9:**
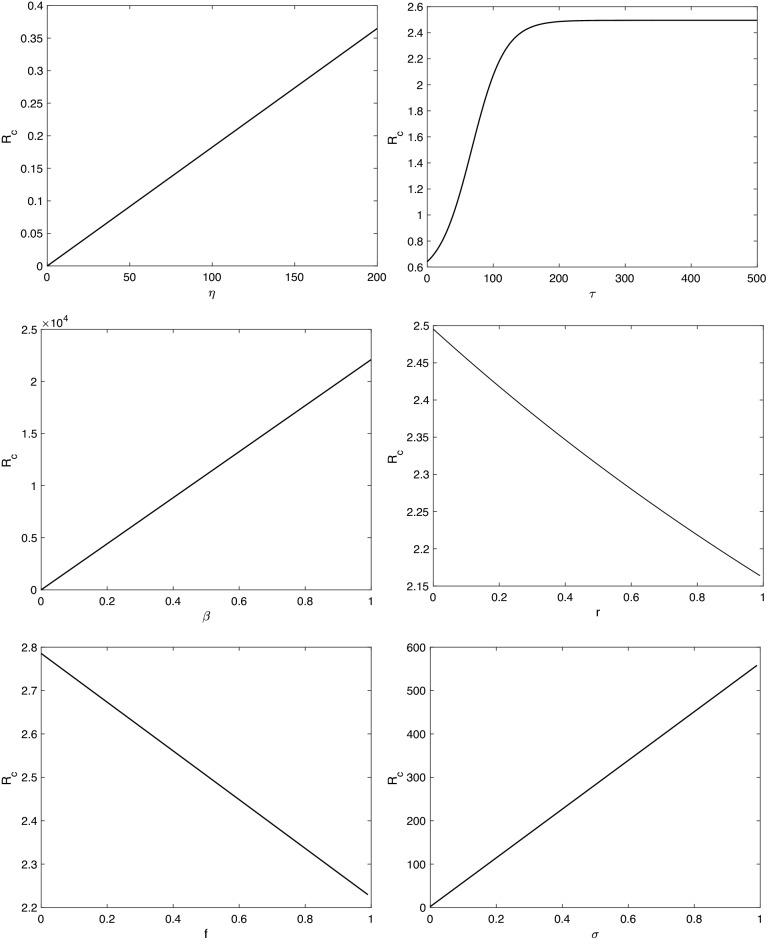
The sensitivity of ${\mathcal {R}}_{c}$ of system (7) with respect to the parameters $\eta ,\ \tau ,\ \beta ,\ r,\ f$, and $\sigma$.

**Figure 10:**
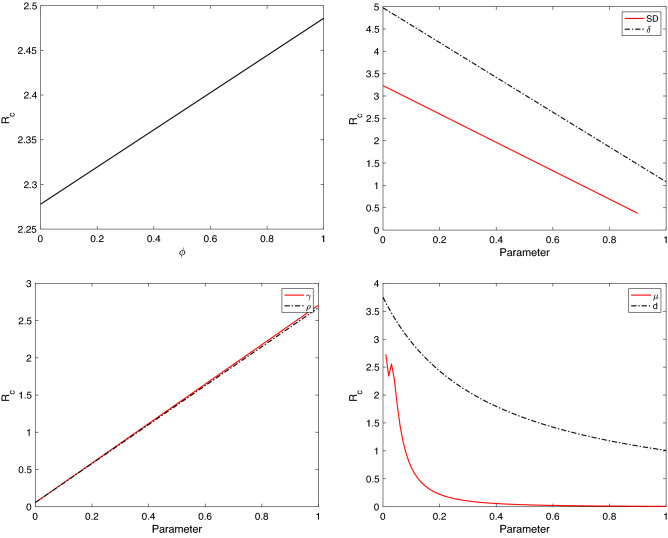
The sensitivity of ${\mathcal {R}}_{c}$ of system (7) with respect to the parameters $\phi ,\ SD,\ \rho ,\ \delta ,\ \gamma ,\ d$, and $\mu$.

Equation (26) shows that increasing some parameters such as $\beta ,\ \gamma$, and $\sigma$ causes an increase in ${\mathcal {R}}_c$. On the other hand, an increase in other parameters, for example, $SD,\ \delta$, and *f*, causes a decrease in ${\mathcal {R}}_c$. The rate of change of ${\mathcal {R}}_c$ corresponding to $\tau ,\ r$, and $\phi$ depends on the value of $\Psi$. If $\Psi$ is greater than one, then this allows ${\mathcal {R}}_c$ to decrease as *r* increases. At the same time, ${\mathcal {R}}_c$ increases as the time delay in vaccine production, $\tau$, and the loss of vaccine immunity rate, $\phi$, increase. Figures 9 and 10 demonstrate the analytic results with $\Psi >1$.

Furthermore, we evaluate the normalized sensitivity index (SI) of ${\mathcal {R}}_c$ with respect to the system’s parameters ${\mathcal {P}}$ by using the formula^25^:

27 $$\begin{aligned} \Gamma _{{\mathcal {R}}_{c}}^{{\mathcal {P}}}=\frac{\partial {\mathcal {R}}_{c}}{\partial {\mathcal {P}}} \frac{{\mathcal {P}}}{{\mathcal {R}}_{c}}. \end{aligned}$$

The sensitivity index estimates the outcome value of ${\mathcal {R}}_c$ when increasing the value of the parameter ${\mathcal {P}}$ by $1\%$. When $\Gamma _{{\mathcal {R}}_c}^{{\mathcal {P}}}$ is positive, then ${\mathcal {R}}_c$ increases with respect to ${\mathcal {P}}$. Contrary, if $\Gamma _{{\mathcal {R}}_c}^{{\mathcal {P}}}$ is negative, then ${\mathcal {R}}_c$ decreases with respect to ${\mathcal {P}}$. Applying the formula in (27), we obtain

$$\begin{aligned} \Gamma _{{\mathcal {R}}_c}^{\eta }&=1,\\ \Gamma _{{\mathcal {R}}_c}^{\delta }&=\frac{-\delta }{(\delta +d+\mu )},\\ \Gamma _{{\mathcal {R}}_c}^{d}&=\frac{-d}{(\delta +d+\mu )},\\ \Gamma _{{\mathcal {R}}_c}^{f}&=\frac{-r e^{-\mu \tau }f(\gamma +\mu )}{\eta \chi },\\ \Gamma _{{\mathcal {R}}_c}^{SD}&=\frac{-\gamma \beta \rho (SD)(\phi +\mu )}{\chi },\\ \Gamma _{{\mathcal {R}}_c}^{\beta }&=\frac{\gamma \beta \rho (1-SD)(\phi +\mu )}{\chi }, \\ \Gamma _{{\mathcal {R}}_c}^{\rho }&=\frac{\gamma \beta \rho (1-SD)(\phi +\mu )}{\chi },\\ \Gamma _{{\mathcal {R}}_c}^{\sigma }&=\frac{r e^{-\mu \tau }\sigma (1-f)(\gamma +\mu )}{\chi }, \\ \Gamma _{{\mathcal {R}}_c}^{\gamma }&=\frac{\mu \gamma \beta \rho (1-SD)(\phi +\mu )}{(\gamma +\mu )\chi }, \\ \Gamma _{{\mathcal {R}}_c}^{\phi }&=\frac{\phi r e^{-\mu \tau }\Big (\gamma \beta \rho (1-SD)-\sigma (1-f)(\gamma +\mu )\Big )}{(r e^{-\mu \tau }+\phi +\mu )\chi }, \\ \Gamma _{{\mathcal {R}}_c}^{r}&=\frac{-r e^{-\mu \tau }(\phi +\mu )\Big (\gamma \beta \rho (1-SD)-\sigma (1-f)(\gamma +\mu )\Big )}{(r e^{-\mu \tau }+\phi +\mu )\chi }, \\ \Gamma _{{\mathcal {R}}_c}^{\tau }&=\frac{\mu \tau r e^{-\mu \tau }(\phi +\mu )\Big (\gamma \beta \rho (1-SD)-\sigma (1-f)(\gamma +\mu )\Big )}{(r e^{-\mu \tau }+\phi +\mu )\chi }, \\ \Gamma _{{\mathcal {R}}_c}^{\mu }&=\frac{-\Big (\gamma \beta \rho (1-SD)\Theta _{1}+r e^{-\mu \tau }\sigma (1-f)(\gamma +\mu )^2\Theta _{2} \Big )}{(\gamma +\mu )(\delta +d+\mu )(r e^{-\mu \tau }+\phi +\mu )\chi }, \end{aligned}$$

**Table 2:** The sensitivity index of ${\mathcal {R}}_{c}$ with respect to the parameters ${\mathcal {P}}$ of system (7).

Parameter ${\mathcal {P}}$	Value	Sensitivity index $\Gamma _{{\mathcal {R}}_{c}}^{{\mathcal {P}}}$
$\eta$	1250	1
$\tau$	120	0.4035
$\beta$	$1.0063\times 10^{-4}$	0.9754
$\rho$	0.85	0.9754
$\gamma$	0.167	0.1885
$\delta$	0.32772	$-0.5417$
*d*	0.23724	$-0.3922$
$\mu$	0.04	$-0.7681$
*SD*	0.3	$-0.4180$
*r*	0.6	$-0.0841$
*f*	0.9	$-0.2216$
$\sigma$	0.0001	0.0246
$\phi$	0.0005	0.001

where $\chi =\gamma \beta \rho (1-SD)(\phi +\mu )+r e^{-\mu \tau }\sigma (1-f)(\gamma +\mu )$. The sensitivity index values for ${\mathcal {R}}_c$ are presented in Table 2, where we have chosen the values of the parameters to satisfy $\Psi >1$. We conclude that increasing the vaccination rate, the efficiency of the vaccine, and the social distancing aids in decreasing the ${\mathcal {R}}_c$. In contrast, increasing the time delay, transmission rate, loss of vaccine immunity rate, and infection after vaccine contributes to the enlargement in ${\mathcal {R}}_c$.

## Conclusion

This paper presented a mathematical model to study the effect of a time delay in vaccine production on the spread of COVID-19. The model was analyzed qualitatively and numerically. Qualitative analysis showed that the system variables are biologically meaningful, bounded, and non-negative. Also, two equilibrium points of the model were discussed: the free equilibrium point, which exists without conditions, and the endemic equilibrium point, which exists provided that the control reproduction number, ${\mathcal {R}}_c$, is greater than unity. Moreover, the formula for the threshold quantity ${\mathcal {R}}_c$ was calculated.

In addition, the local stability of equilibrium points was investigated. The free equilibrium point is locally asymptotically stable if ${\mathcal {R}}_c<1$ and the time delay, $\tau$, is greater than or equal to a critical value ($\tau ^*$). In contrast, when the time delay is less than $\tau ^*$, the stability of the free equilibrium changes, and a Hopf bifurcation occurs. Furthermore, the conditions for the stability of the endemic equilibrium were discussed.

The numerical analysis showed agreement with the results of the qualitative analysis. Also, the effect of the vaccine’s time delay, vaccine rate, and efficiency on the model dynamics was studied. Finally, the sensitivity analysis was delivered for ${\mathcal {R}}_c$.

The model analysis revealed the significance of raising the vaccination rate while reducing the vaccine loss immunity rate. However, if vaccines are not highly effective, raising the vaccination rate will not lower the size of the infected class.

## Supplementary Information

Supplementary Information.

## Data Availability

All data generated or analysed during this study are included in this published article.
