# Supplementary material for: A time-delayed model for the spread of COVID-19 with vaccination
Source: Sci Rep. 2022 Nov 13;12:19435. doi: 10.1038/s41598-022-23822-5 (PMC9659561; doi:10.1038/s41598-022-23822-5)
Supplement: Supplementary file 1 — Supplementary Information. [file 41598_2022_23822_MOESM1_ESM.pdf]

A Time-Delayed Model for the Spread of COVID-19 with Vaccination

Salma M. Al-Tuwairqi* and Sara K. Al-Harbi

Mathematics department, King Abdulaziz University, Jeddah, Saudi Arabia
*email: saltuwairqi@kau.edu.sa

Supplementary Material

The MATLAB Code for the model is demonstrated below.

```
function yp = Model3(t,y,Z)
global eta mu beta gamma delta death sigma1 sigma2 r1 r2 f1 f2 alpha1
   alpha2 phi1 phi2 ro SD tau

ylag1 = Z(:,1); % ylag1(1) for S(t-tau); ylag1(3) for I(t-tau)

yp = zeros(5,1);
if t<=tau   %Before the vaccine is available
    yp(1)=mu-beta*ro*(1-SD)*(eta/mu).*y(1).*y(3)-r1*exp(-mu*tau).*
       ylag1(1)+phi1.*y(5)-mu.*y(1);
    yp(2)=beta*ro*(1-SD)*(eta/mu).*y(1).*y(3)-(gamma+mu).*y(2);
    yp(3)=gamma.*y(2)+sigma1*(1-f1)*(eta/mu).*y(3).*y(5)-(delta+death+
       mu).*y(3);
    yp(4)=delta.*y(3)-alpha1*delta*exp(-mu*tau).*ylag1(3)-mu.*y(4);
    yp(5)=r1*exp(-mu*tau).*ylag1(1)+alpha1*delta*exp(-mu*tau).*ylag1
       (3)-sigma1*(1-f1)*(eta/mu).*y(3).*y(5)-(phi1+mu).*y(5);
else
    yp(1)=mu-beta*ro*(1-SD)*(eta/mu).*y(1).*y(3)-r2*exp(-mu*tau).*
       ylag1(1)+phi2.*y(5)-mu.*y(1);
    yp(2)=beta*ro*(1-SD)*(eta/mu).*y(1).*y(3)-(gamma+mu).*y(2);
    yp(3)=gamma.*y(2)+sigma2*(1-f2)*(eta/mu).*y(3).*y(5)-(delta+death+
       mu).*y(3);
    yp(4)=delta.*y(3)-alpha2*delta*exp(-mu*tau).*ylag1(3)-mu.*y(4);
    yp(5)=r2*exp(-mu*tau).*ylag1(1)+alpha2*delta*exp(-mu*tau).*ylag1
       (3)-sigma2*(1-f2)*(eta/mu).*y(3).*y(5)-(phi2+mu).*y(5);
end
end
```

```
% Experiment 1: Stability of COVID-19 free equilibrium
clear; clc
```

```
global eta mu beta gamma delta death sigma1 sigma2 r1 r2 f1 f2 alpha1
    alpha2 phi1 phi2 ro SD tau
%% Parameters values
eta=30;  mu=0.07;  beta=0.005; gamma=0.167;
delta=0.32772;  death=0.23724;  ro=0.85; SD=0.60;
r1=0;   sigma1=0;     f1=0;   alpha1=0;      phi1=0;
r2=0.5; sigma2=0.001; f2=0.7; alpha2=0.0001; phi2=0.005;
tau=120;
t=[0 300];
%% ICs:
S1=0.8; E1=0.1; I1=0.05; R1=0;  V1=0;
S2=0.6; E2=0.2; I2=0.15; R2=0;  V2=0;
S3=0.4; E3=0.3; I3=0.2;  R3=0;  V3=0;
sol1=dde23('Model3',tau,[S1;E1;I1;R1;V1],t);
sol2=dde23('Model3',tau,[S2;E2;I2;R2;V2],t);
sol3=dde23('Model3',tau,[S3;E3;I3;R3;V3],t);
%% COVID-19 free equilibrium
DFE_S0=(eta*(phi2+mu))/(mu*(phi2+mu+r2*exp(-mu*tau)))*(mu/eta);
DFE_V0=(eta*r2*exp(-mu*tau))/(mu*(phi2+mu+r2*exp(-mu*tau)))*(mu/eta);

DFE=[DFE_S0,0,0,0,DFE_V0]
IC1=[sol1.y(1,end),sol1.y(2,end),sol1.y(3,end),sol1.y(4,end),sol1.y(5,
    end)]
IC2=[sol2.y(1,end),sol2.y(2,end),sol2.y(3,end),sol2.y(4,end),sol2.y(5,
    end)]
IC3=[sol3.y(1,end),sol3.y(2,end),sol3.y(3,end),sol3.y(4,end),sol3.y(5,
    end)]
%% Plot
figure(1)
pp1=plot(sol1.x,sol1.y(1,:),'b-',sol2.x,sol2.y(1,:),'r--',sol3.x,sol3.
    y(1,:),'k-.','LineWidth', 1);
xlabel(' Time (days) ')
ylabel(' Susceptible individuals ')
set(gca,'FontSize',15)
legend('IC 1','IC 2','IC 3');
figure(2)
pp2=plot(sol1.x,sol1.y(2,:),'b-',sol2.x,sol2.y(2,:),'r--',sol3.x,sol3.
    y(2,:),'k-.','LineWidth', 1);
xlabel(' Time (days) ')
ylabel(' Exposed individuals ')
set(gca,'FontSize',15)
legend('IC 1','IC 2','IC 3');
figure(3)
pp3=plot(sol1.x,sol1.y(3,:),'b-',sol2.x,sol2.y(3,:),'r--',sol3.x,sol3.
    y(3,:),'k-.','LineWidth', 1);
xlabel(' Time (days) ')
ylabel(' Infected individuals ')
```

```
set(gca,'FontSize',15)
legend('IC 1','IC 2','IC 3');
figure(4)
pp4=plot(sol1.x,sol1.y(4,:),'b-',sol2.x,sol2.y(4,:),'r--',sol3.x,sol3.
    y(4,:),'k-.','LineWidth', 1);
xlabel(' Time (days) ')
ylabel(' Recovered individuals ')
set(gca,'FontSize',15)
legend('IC 1','IC 2','IC 3');
figure(5)
pp5=plot(sol1.x,sol1.y(5,:),'b-',sol2.x,sol2.y(5,:),'r--',sol3.x,sol3.
    y(5,:),'k-.','LineWidth', 1);
xlabel(' Time (days) ')
ylabel(' Vaccinated individuals ')
set(gca,'FontSize',15)
legend('IC 1','IC 2','IC 3');
```
